# A New Medical Society

**Published:** 1846-02

**Authors:** 


*A New Medical Society.*—We have before us a circular signed by a number of Medical gentlemen, residing in the Rock River Valley, calling a convention of Physicians at Rockford on the 17th of February, for the purpose of organizing a Medical Society. It is much to be regretted that the notice is so short as to exclude many who would be happy to join in the movement. We hope in our next number to have the proceedings of the convention to lay before our readers, when our remarks will be more extended.—[Ed.

